# Identification of Fungi Involved in Onychomycosis in Patients of a Spanish Rural Area

**DOI:** 10.3390/jof7080623

**Published:** 2021-07-31

**Authors:** Felix Marcos-Tejedor, Marta Mota, María José Iglesias-Sánchez, Raquel Mayordomo, Teresa Gonçalves

**Affiliations:** 1 Department of Medical Sciences, Faculty of Health Sciences, University of Castilla-La Mancha, 45600 Talavera de la Reina, Spain; 2 Faculty of Medicine, University of Coimbra, 3004-504 Coimbra, Portugal; martamota@ipocoimbra.min-saude.pt (M.M.); tmfog@ci.uc.pt (T.G.); 3 CNC—Center for Neuroscience and Cell Biology, University of Coimbra, 3004-504 Coimbra, Portugal; 4 Department of Medical and Surgical Therapy, University Center of Plasencia, University of Extremadura, 10600 Plasencia, Spain; iglesiasmjo@gmail.com; 5 Department of Anatomy and Human Embryology, University Center of Plasencia, University of Extremadura, 10600 Plasencia, Spain; rmayordo@unex.es

**Keywords:** onychomycosis, diagnosis, PCR, Fungus, nails

## Abstract

Onychomycosis is one of the most frequent reasons for visiting podiatrist clinics. Complementary tests and the accurate identification of the infectious agents are key issues for a successful treatment of onychomycosis. This is particularly important when lifestyle, age and immunodepressed patients increase the prevalence of non-dermatophyte fungal infection. In this paper, we describe issues related to onychomycosis prevalence in a population of patients, mostly with rural lifestyles, visiting a podiatry clinic in a rural area of Spain. A total of 51 cases were studied with an average age of 65.96 ± 21.28 years (the youngest being 16 years and the oldest being 95 years). Fungal agents were isolated using conventional sampling and microbiological culture techniques. The results obtained with these techniques were compared with the results obtained with a direct methodology using molecular biology, by PCR and nucleotide sequencing of the ITS-5.8S rDNA fragment. The classical culture methodology confirmed the infection in 76.5% of the samples (*n* = 39), while the PCR confirmed the infection in 84.3% (*n* = 51) of the nails, although the difference between these results did not show statistical significance (*p* = 0.388). We found a high variability in agents, with more yeasts than dermatophytes as etiological agents of onychomycosis. However, only among individuals older than 65 years, was the difference between yeasts (82%) and dermatophytes (18%) was statistically significant (*p* = 0.004). Among the agents of non-dermatophyte onychomycosis, we found predominantly fungi (yeasts) of the *Candida* genus, interestingly with no isolates of *Candida albicans*, and moulds of the *Aspergillus* genus.

## 1. Introduction

Onychomycosis is a fungal infection of the nail with a global prevalence of 5.5–8% and a higher prevalence in the first toenail [1,2]. New lifestyles, advanced age, obesity, and immunosuppression states, such as diabetes mellitus, organ transplants, the use of corticosteroids and antineoplastic drugs, are increasing the prevalence of infections by yeasts, moulds and non-dermatophyte fungi (NDO) [3,4,5].

Complementary tests are necessary to accurately diagnose onichomycosis and to gather epidemiological data to study the evolution of the etiological agents. Some pathologic abnormalities lead to signs and symptoms, such as yellowish nail, onycholysis or leukonychia, similar to onychomycosis, such as onychodystrophy which is originated by psoriasis, lichen planus, traumatisms, some physiologic changes associated with aging, and other unusual syndromes [1,2]. Consequently, a wrong clinical diagnosis is often the cause of onychomycosis treatment failure, since this infection only accounts for 50–68% of all nail disorders. Moreover, the correct knowledge of the infectious agent provides guidance to the appropriate treatment and minimizes possible toxic effects of the drugs used [3,6,7,8,9].

Onychomycosis is not self-limited and does not resolve spontaneously. Early treatment of onychomycosis is important, since this pathology can restrict everyday activities. It is associated with negative psychosocial impact that leads patients to isolation and demands effective treatments [3,10,11]. In the elderly, a target population for onychomycosis, it can have acute presentations with inflammatory response and lesions, ultimately increasing opportunistic subcutaneous bacterial infections [12]. Therefore, besides proper clinical knowledge, the availability of laboratory tests to confirm the infection and to identify the agent is of the utmost importance [1,6,7,13]. The most consensual technique for the laboratory diagnosis of onychomycosis includes direct light microscopy for the identification of fungal elements, using a fresh preparation with 20% KOH; microbiological culture; identification of the agent by the microscopic study of fungal morphology. However, this approach has limitations. The microbiological methods have the disadvantage that cultures may lead to 40% false-negatives, it is time consuming and requires 2 to 4 weeks for definitive results; KOH studies result in 20% false-negative cases; the differentiation of the morphological structures discriminatory between species failed to be formed [1,6,14,15].

Based on previous studies, we aimed to detect fungi in toenail samples using the polymerase chain reactions (PCR) and to compare the results obtained by this molecular biology technique with the results obtained by using the classical conventional culture and microscopic morphological identification. The molecular biology approach increased the confirmation of nail infections, while reducing the time needed for the confirmation of the diagnosis of onychomycosis [14,16,17,18,19]. We describe issues related to the epidemiology of onychomycosis prevalence in a rural population of patients attending a podiatry clinic by detecting genetic material of fungi directly in toenail samples suspected of having onychomycosis.

## 2. Materials and Methods

### 2.1. Patients and Sample Collection

This study was conducted following the guidelines of the Declaration of Helsinki, whilst the handling of human samples was approved by the Bioethical Commission of the Universidad de Extremadura (ref 35/2012). Before the collection of samples, all the participants gave their verbal and written consent.

Samples of convenience consisted of pieces of toenail clippings from 51 patients with clinical (interview and exploration) suspicion of having onychomycosis in the first toe. These patients were assisted in a podiatric clinic at the North of Extremadura and the South of Salamanca, a rural area of Spain. To avoid environmental contamination of the samples, before taking the sample, the patients’ fingers were irrigated with 70° alcohol, and nail clippings were taken after the complete evaporation of the disinfectant.

### 2.2. Microbiological Culture

One piece of each toenail sample was cultured on Sabouraud dextrose agar plates with chloramphenicol (Condalab, Spain), a selective method for the isolation of fungi, at 30 °C during three to four weeks, or until positive for fungal growth.

### 2.3. Molecular Analysis

Using a small fragment of the same toenail sample, total DNA was extracted using InstaGene MatrixTM (Bio-Rad, Algés, Portugal), following the manufacturer’s instructions. The extracted DNA concentration and purity were measured by a Nanodrop 2000 (Thermo Fisher Scientific, Wilmington, DE, USA), by the ratio of absorbances at 260 to 280 nm (A260/A280).

Conventional PCR assays were carried out in a 50 µL solution with 25 μL of Master Mix (NZYTech), 1–5 μL of sample DNA and 0.5 μL of each primer (20 µM). The universal oligonucleotides used were ITS1 [5′ TCCGTAGGTGAACCTGCGG3′] and ITS4 [5′ TCCTC CGCTTATTGATATGC 3′], amplifying a sequence located in an internal transcriber spacer region of ribosomal DNA ITS-5.8S. The thermal program was: 95 °C for 5 min, followed by 35 cycles at 95 °C for 30 s, annealing 56 °C for 60 s, with a final extension at 72 °C for 60 s and finally at 72 °C for 7 min.

A Real-Time PCR assay (RT-PCR) was performed to further check the samples with negative results in the conventional PCR test, as a strategy to increase the sensitivity of the amplification. This RT-PCR test was performed using LightCycler 2.0 (Roche, Basel, Switzerland). The PCR reactions were prepared in a total volume of 20 μL (2–5 µL of sample DNA, 0.8 µM of each primer, 4 µL of LightCycler FastStart DNA Master Plus SYBR Green I (Roche, Basel, Switzerland) and water). RT-PCR test conditions were: 95 °C for 10 min, followed by 45 cycles at 95 °C for 10 s, 56 °C for 5 s, with a final extension at 72 °C for 24 s, and finally, cycles at 40 °C for 30 s.

The results of conventional PCR tests and positive results of the RT-PCR tests were visualised in a 1.5% agarose electrophoresis gel stained with ethidium bromide (Sigma-Aldrich^®^ Solutions, Saint louis, MI, USA) for 30 min at 120 V and visualized with ultraviolet light (Transilluminator^®^-UVP). The bands corresponding to fungal DNA were cut, purified using the Kit NucleoSpin^®^ Gel and PCR Clean-Up (Macherey-Nagel, Dylan, Germany) and sent for nucleotide sequencing elsewhere (LGC Genomics GmbH). The identification was obtained by comparing the sequences in the NCBI database using a Blast search.

### 2.4. Confocal Fluorescence Microscopy

Confocal fluorescence microscopy was performed to visualize fungal structures in the nail samples. The toenail samples were immersed in 50% KOH until reaching full degradation. Afterwards, the samples were stained with Evans blue and Calcofluor white for 15–20 min. Confocal microscopy images were obtained with the Carl Zeiss Axio Observer Z1 fluorescence microscope, with Plan-ApoChromat 20× and 63×/1.40 immersion objectives, using Zeiss Zen lite and Image J software to analyse the images.

### 2.5. Statistics

The SPSS^®^ v24 program was used for the statistical study of the results, with a statistical significance of 5% (*p* < 0.05).

## 3. Results

**Figure 1:**
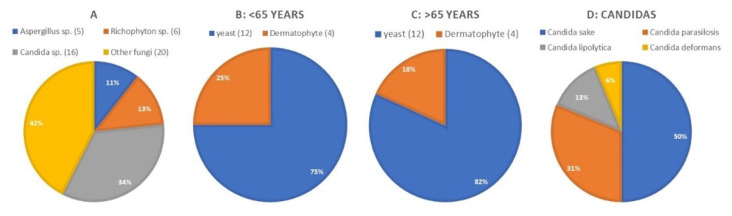
Details of the patterns of distribution of infecting agents. (**A**) Genera of fungi with the highest prevalence. (**B**) Dermatophytes and yeast in patients with less than 65 years. (**C**) Dermatophytes and yeast in patients over 65 years old. (**D**) Yeast species isolated.

The nail samples were obtained from a population of patients from the region of North of Extremadura and the South Salamanca. The average age of the patients was 65.96 ± 21.28 years (with a range of 16 to 95 years). Concerning gender, 29 women and 22 men were enrolled in this study (Table 1). Among the 51 patients suspected of having fungal infections in the toe nail, 76.5% (*n* = 39) tested positive in the microbiological culture, in 48.8% (*n* = 19) of the patients with positive culture, the nail infection was caused by dermatophyte, and 25.6% (*n* = 10) the infectious agents were yeasts. In some cases, 25.6% (*n* = 10), the infection was mixed, including a dermatophyte and a yeast (Figure 1).

Using conventional PCR, fungal DNA was detected in 58.8% (*n* = 30) of the samples with positive results, but not detected in 41.2% (*n* = 21). The samples that tested negative using the conventional PCR were analysed by RT-PCR. With this methodology, positive results increased to 61.9% (*n* = 13) of the samples while 38.1% (*n* = 8) continued negative. It can be considered that, using molecular analysis (conventional PCR and Real-time PCR), the number of positive results was 43 out of 51 (84.3%), while the number of negative results was 8 (15.7%) (Table 2).

When we compared the results obtained using PCR (plus RT-PCR) with the microbiological cultures, there was a match in 76.5% of the cases (Table 2). The results were discordant in 23.5% (*n* = 12) of the samples, 15.7% (*n* = 8) with positive PCR but with no growth in culture and 7.8% (*n* = 8) of the samples with positive culture resulted in negative PCR detection of fungal DNA. The statistical study of the results obtained with the two different techniques, using the McNemar test, did not show a statistical significative difference (*p =* 0.388).

A high number of agents was found, with more yeasts than dermatophytes involved in onychomycosis. Using Fisher’s exact test, we found a significant statistical difference (*p =* 0.004) only among individuals over 65 years. This difference between yeasts (82%) and dermatophytes (18%) can be found both in women and men.

Of the 43 samples that tested positive in the PCR test, only the PCR products of 38 samples were sequenced, revealing 31 yeast isolates, 10 isolates of filamentous fungi and 8 dermatophytes (Table 1 and Figure 1). The yeast species found were predominantly of the *Candida* genus, including five isolates of *Candida parapsilosis* and eight isolates of *Candida sake*. Also, among the agents of non-dermatophyte onychomycosis (NDO), we found moulds of the genus *Aspergillus*.

**Figure 2:**
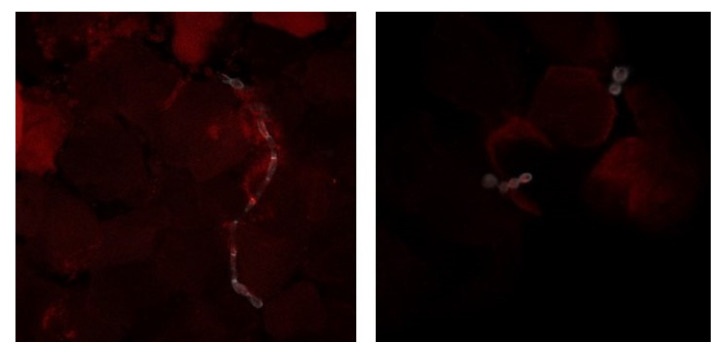
Confocal fluorescence microscopy of nail samples. The nails were treated with KOH and stained with Evans-blue and calcofluor. The images are illustrative of the samples studied by this technique. On the left side picture, a dermatophyte infecting the nail can be seen and on the right yeast forms in the nail material can be seen (20× and 63×/1.40 immersion objectives).

Finally, we focused on the four cases that tested positive in the culture (two cases positive for yeast and two for dermatophytes) and negative in the PCR assays. These samples were studied using confocal fluorescence microscopy to confirm the discordant results obtained. This technique allowed the observation of fungal structures in the nail material, and consequently to confirm the positive culture (Figure 2).

## 4. Discussion

In this study, we describe that 92.2% of the nail alterations in the studied rural population were due to fungi; the remaining were due to onycopathies with other origins. These results are different from those described by other authors. According to the available literature, 50–68% of nail alterations are originated by fungi. This discrepancy may arise from clinical aspects, if the patients were not approached by an expert in nail alterations [6,7,8,9], due to the fact that, in our study, several techniques were used to make confirmatory assays of the presence of fungi in the nails exhibiting abnormalities. Our results also support the data obtained by others showing that onychomycosis mainly affects the nail of the first toe [1,2].

In respect to the fungal agents involved, we found that the nail damage is mainly caused by yeast infections and NDO. The literature indicates that *T. rubrum* is the most prevalent agent of onychomycosis. Nevertheless, as described previously, there are populations, such as immunosuppressed patients, and lifestyles, in whom there is an increasing prevalence of other fungi, with a special increase in infections due to *Aspergillus* sp. [1,4,5,15]. The population under study in the present work were predominantly over 65 years old, living in rural areas and engaged in agriculture and livestock, while the majority of the youngest subjects were immunosuppressed (HIV, Leukemia and renal transplant. A careful look at the results shows that the yeasts, especially *Candida* spp., were more common than dermatophytes. *T. interdigitale* was more common than *T. rubrum* in dermatophytic onychomycosis. Previous studies carried out in urban areas of Spain revealed that dermatophytes and *T. rubrum* were the most common etiological agents of onychomycosis [16,20,21]. We believe that the discrepancies between our findings and those others are due to different lifestyles, rural vs. urban.

Using PCR, we were able to detect infections in 84.3% of the samples, while the cultures accounted for 76.5% positive results. There was 68.62% match between the positive results by both techniques (culture vs. molecular biology) and 7.8% between negative results. These findings are similar to our previous work and the results obtained by other authors [6,14,16].

Despite the 23.5% discrepancy in the total results, the statistics did not result in any difference between the results found with the two groups of techniques (*p =* 0.388), revealing that the PCR tests provide the same results as the reference method but in a shorter period of time (few hours), while microbiological cultures require from two to four weeks and can result in 40% false negatives [1,2,14]. The results obtained here seem to indicate that PCR can be a confirmatory test to identify onychomycosis agents.

In the present work, we believe that the diagnosis of onychomycosis can take advantage of PCR testing directly from the toenail, revealing higher prevalence of onychomycosis caused by other NDOs, because previous works, using conventional assays, only detected dermatophyte infections [14,15,17].

We did not expect that samples testing positive in the culture would result in negative PCR tests. For this reason, we focused on these four samples and performed confocal fluorescence microscopy that allowed us to confirm the presence of fungal structures in those nails. We hypothesized that the lack of detection of fungal DNA by PCR could be due to the inhibition of the polymerase chain reaction, because these four results were obtained from multiple nails (10.81 ± 7.33 mg) with a high amount of extracted DNA (276 ± 304.74 ng/µL) or because of impurities (mean ratio of the 260/280 absorbance was 1.3 ± 0.11). We performed successive dilutions of the DNA with no success in the PCR (resulys not shown), leading us to conclude that the inhibition was due to other factors, as reported by others [22,23].

Nowadays, molecular methods are the solid base of diagnostic tests for the medical clinic practice. Because of this, the accessibility to databases of biological sequences is increasingly easier, involving the continuous development of molecular biology. These techniques are increasingly used in the diagnosis of fungal infection with an improvement in sensitivity and specificity. However, these are still not used routinely in the diagnosis of onychomycosis in clinical practice [1,2,6,18,19].

## 5. Conclusions

We can conclude that, nowadays, in rural areas, there is a higher prevalence of onychomycosis caused by non-dermatophytes fungi, mainly in the population older than 65.

The application of PCR techniques improves the diagnosis of onychomycosis, especially with regards to the reduction of the time needed for the diagnosis confirmation, as well as the detailed detention of the infectious agent.

## Figures and Tables

**Table 1:** Characterization of the population under study, detection of fungal infection in the nails and identification of the fungal agents.

Subjets	Years	Gender	Culture	PCR	Infecting Agents	Microscopical Findings **
**1**	60	Female	Positive	Positive	*Trichophyton interdigitale* *Cladosporium* sp. *Pichia* sp.	
**2**	77	Female	Positive	Positive	*Microsporum incurvatum*	
**3**	57	Female	Positive	Positive	*Candida lipolytica* *Candida sake* *Penicillium* sp. *Kazachstania* sp.	
**4**	45	Female	Positive	Positive	*Rhodotorula mucilaginosa*	
**5**	68	Female	Negative	Negative		
**6**	67	Female	Negative	Positive	*Aspergillus ruber* *Debarymyces hansenii*	
**7**	88	Male	Positive	Positive	*Pichia kluyveri*	
**8**	23	Male	Negative	Negative		
**9**	81	Male	Positive	Positive	*Pichia membranaefaciens*	
**10**	75	Female	Positive	Positive	*Trichophyton interdigitale*	
**11**	75	Female	Positive	Positive	*Candida parapsilosis*	
**12**	31	Female	Positive	Positive	*Trichophyton rubrum*	
**13**	55	Female	Positive	Positive	*Candida parapsilosis*	
**14**	53	Female	Positive	Positive	*Trichophyton interdigitale*	
**15**	80	Male	Negative	Positive	*Trichosporon dermatis*	
**16**	55	Female	Positive	Positive	*Candida parapsilosis*	
**17**	80	Male	Positive	Positive	*Candida sake*	
**18**	75	Female	Positive	Positive	*Pichia* sp. *Epicoccum nigrum*	
**19**	85	Female	Negative	Positive	*Candida sake*	
**20**	79	Male	Positive	Positive	NP *	
**21**	50	Female	Positive	Positive	*Aureobasidium pullulans*	
**22**	84	Male	Positive	Positive	*Phoma herbarum*	
**23**	86	Female	Positive	Positive	NP *	
**24**	79	Male	Positive	Positive	*Cryptococcus victoriae*	
**25**	82	Female	Positive	Positive	NP *	
**26**	42	Male	Positive	Positive	*Cryptococcus diffluens*	
**27**	65	Male	Positive	Positive	*Trichophyton rubrum*	
**28**	32	Male	Negative	Positive	*Trichosporon asahii*	
**29**	22	Female	Positive	Positive	*Candida lipolytica*	
**30**	45	Female	Positive	Positive	*Candida sake*	
**31**	91	Male	Positive	Positive	*Aspergillus flavus*	
**32**	80	Male	Positive	Positive	*Candida parapsilosis*	
**33**	85	Female	Positive	Positive	*Aspergillus sydowii*	
**34**	78	Female	Positive	Positive	*Candida parapsilosis Rhodotorula mucilaginosa*	
**35**	65	Female	Positive	Positive	*Candida sake* *Candida deformans*	
**36**	67	Female	Positive	Positive	*Candida sake*	
**37**	95	Female	Positive	Positive	*Kazachstania* sp.	
**38**	83	Male	Positive	Positive	NP *	
**39**	89	Female	Negative	Positive	*Candida sake*	
**40**	81	Female	Negative	Positive	*Cryptococcus uniguttulatus* *Trichophyton interdigitale*	
**41**	52	Male	Positive	Negative		Presence of dermatophyte
**42**	88	Male	Positive	Negative		Presence of yeast
**43**	56	Female	Negative	Negative		
**44**	76	Male	Positive	Negative		Presence of dermatophyte
**45**	80	Female	Negative	Positive	NP *	
**46**	30	Male	Negative	Positive	*Candida sake*	
**47**	69	Male	Positive	Positive	*Epicoccum nigrum*	
**48**	16	Female	Negative	Negative		
**49**	85	Male	Positive	Negative		Presence of yeast
**50**	84	Male	Positive	Positive	*Cryptococcus diffluens*	
**51**	18	Male	Positive	Positive	*Trichophyton rubrum* *Aspergillus sydowii*	
**Mean**	65.96 ± 21.28					

* NP—It was not possible to sequence the genetic material to determine the infecting agent. ** Fluorescence microscopy as described under Materials and Methods.

**Table 2:** Analysis of the relations between the results obtained by PCR and culture.

	Culture		
PCR	Negative % (*n*)	Positive % (*n*)	Total % (*n*)
Negative % (*n*)	7.8% (4)	7.8% (4)	15.7% (8)
Positive % (*n*)	15.7% (8)	68.6% (35)	84.3% (43)
**Total % (*n*)**	23.5% (12)	76.5% (39)	100% (51)
